# Crucial roles of different RNA-binding hnRNP proteins in Stem Cells

**DOI:** 10.7150/ijbs.55120

**Published:** 2021-02-08

**Authors:** Wen Xie, Hecheng Zhu, Ming Zhao, Lei Wang, Shasha Li, Cong Zhao, Yao Zhou, Bin Zhu, Xingjun Jiang, Weidong Liu, Caiping Ren

**Affiliations:** 1 Cancer Research Institute, Department of Neurosurgery, School of Basic Medical Science, Xiangya Hospital, Central South University, Changsha 410008, China.; 2 Changsha Kexin Cancer Hospital, Changsha, Hunan 410205, China.; 3 The Key Laboratory of Carcinogenesis of the Chinese Ministry of Health and the Key Laboratory of Carcinogenesis and Cancer Invasion of the Chinese Ministry of Education, Central South University, Changsha 410008, China.; 4 Department of Neurosurgery, Xiangya Hospital, Central South University, Changsha 410008, China.

**Keywords:** Heterogeneous nuclear ribonucleoprotein, Stem cell, mRNA stability, Epigenetic regulation, Telomere length and telomerase activity

## Abstract

The self-renewal, pluripotency and differentiation of stem cells are regulated by various genetic and epigenetic factors. As a kind of RNA binding protein (RBP), the heterogeneous nuclear ribonucleoproteins (hnRNPs) can act as “RNA scaffold” and recruit mRNA, lncRNA, microRNA and circRNA to affect mRNA splicing and processing, regulate gene transcription and post-transcriptional translation, change genome structure, and ultimately play crucial roles in the biological processes of cells. Recent researches have demonstrated that hnRNPs are irreplaceable for self-renewal and differentiation of stem cells. hnRNPs function in stem cells by multiple mechanisms, which include regulating mRNA stability, inducing alternative splicing of mRNA, epigenetically regulate gene expression, and maintaining telomerase activity and telomere length. The functions and the underlying mechanisms of hnRNPs in stem cells deserve further investigation.

## Introduction

Stem cells are characterized by self-renewal and the potential of multidirectional differentiation. In 1963, hematopoietic stem cells were found in the spleen, which pioneered a field for stem cell research 1, 2. From then on, the study of stem cells has attracted more and more attention. In mammals, there are two types of stem cells, embryonic stem cells and adult stem cells. Embryonic stem cells (ESCs) are derived from blastocyst, which are developmentally pluripotent and can theoretically differentiate into almost all types of cells and organs of an organism. Adult stem cells (ASCs) exist in various tissues, which include hematopoietic stem cells, neural stem cells, mesenchymal stem cells, and retinal stem cells. ASCs also have some differentiation capacity and can be directed to differentiate into specific types of cells. According to their differentiation potential, stem cells can be divided into totipotent stem cells, pluripotent stem cells, multipotent stem cells, and unipotent stem cells. In view of the multidirectional potential of stem cells, researchers regard them as the seeds of cells, tissues and organs for replacement therapy. However, the most commonly used cell lines for stem cell research are not embryonic stem cells, but reprogrammed induced pluripotent stem cells (iPS cells). Through lentiviral transfection, transcription factors (such as Oct4, Sox2 and Nanog) are transferred into mammalian adult cells to dedifferentiate the adult cells into pluripotent stem cells, which are known as iPS cells with embryonic stem cell characteristics. iPS cells can reduce ethical controversy in clinical practice. In recent years, stem cell researches have obtained great successes in organ transplantation 3, treatment of tumors 4, and new drug development 5. But due to the self-renewal capacity of stem cells, they act as a double-edged sword, which can also be transformed into malignant tumor. Therefore, the biological process of stem cells is needed to be further explored, and such exploration may bring innovative ideas for treatment of relevant diseases.

RNA-binding proteins (RBPs) are a unique class of proteins in eukaryotic cells, which interact with RNA via specific RNA-binding domains (RBDs). A single RBP functions in cells by binding to multiple RNA sequences. RBP binds to target genes through recognizing the specific nucleotide sequence of the open reading frame (ORF) or untranslated region (UTR) in their transcripts, mainly through different RBDs 6, such as RRM, K homology (KH) domain, double-stranded RNA-binding base sequence (dsRBM), zinc finger (ZF) domain, etc. 7. However, some RBPs do not bind with RNA through RBD, but rather by affinity distribution and synergistic interactions with other effectors 8. Recent studies indicated that RBP can affect the alternative splicing, transcription, stability and intracellular localization of bound mRNAs to change their functions, and thus participate in the self-renewal and differentiation of stem cells 9.

hnRNPs are one of the most typical kinds of RNA-binding proteins 10. hnRNP family include hnRNPs A-U and several other RNA-binding proteins in eukaryotic cells. Most of hnRNPs are mainly located in the nucleus, while a few of them present in both the nucleus and cytoplasm 11. hnRNPs can regulate mRNA splicing 12 and stability 13, gene transcription and translation 14, DNA damage repair, and telomere length maintenance 15. As nucleoplasmic shuttle proteins, they play significant roles in cell signaling processes. It has been found that hnRNPs not only serve as therapeutic targets and prognostic biomarkers in tumors, but also play important roles in stemness maintenance and differentiation of stem cells.

In recent years, some reviews have illustrated how hnRNPs regulate the biological function of cells 15 and their roles in the occurrence and development of relevant diseases 16. For example, in the previous reviews, it was pointed out that hnRNPs can recruit lncRNAs to enrich them in the nucleus, regulate the alternative splicing of lncRNAs, and change genome structure, etc. 9, 13.

In this review, we mainly discuss the roles of hnRNPs and how they function in biological processes of stem cells.

## The structure and function of hnRNPs

**Figure 1:**
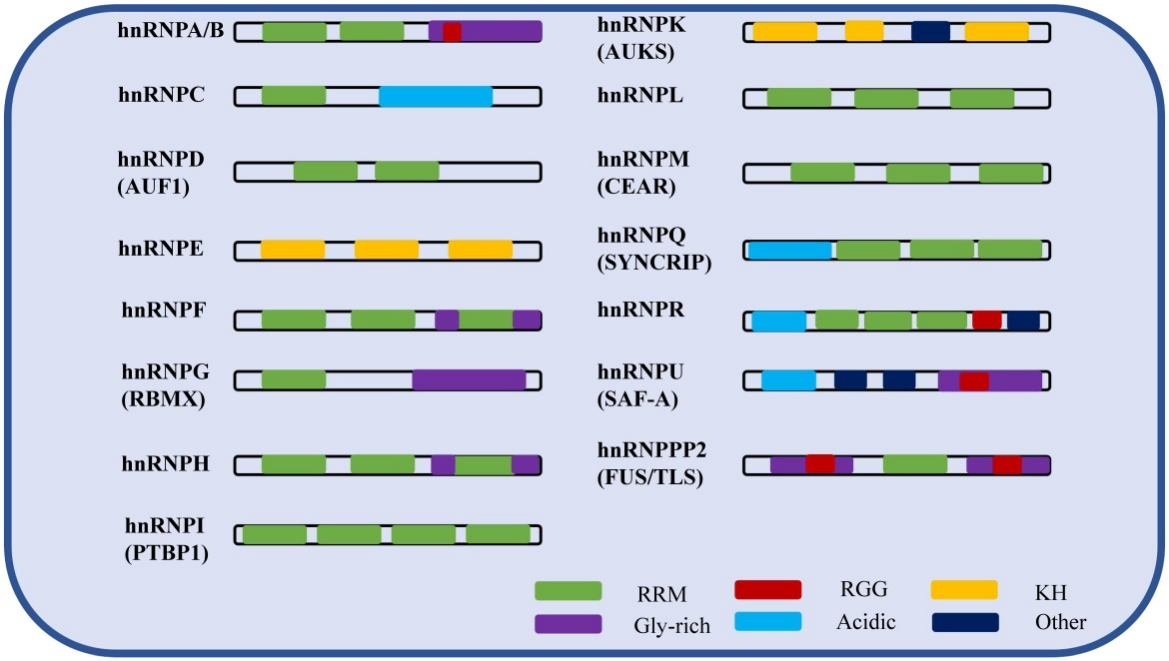
The schematic of the hnRNP domains.

hnRNPs are nucleic acid metabolism family of histones that bind with transcripts produced by RNA polymerase II in the nucleus, and are involved in processes such as nucleic acid metabolism, cell differentiation, and apoptosis 11. Among them, hnRNP A/B and C were first separated from the complex by biochemical methods (sucrose density gradient separation method) 16. Subsequently, due to the RNA-binding specificity of the proteins, other hnRNP family member complexes were successfully identified in intact cells by using UV cross-linking, with about 20 species from A-U 17. hnRNPs are typical RNA-binding and modular proteins, mainly composed of RNA binding motifs and auxiliary domains. The RNA binding motifs include RRM domains, RGG domains 18 and KH domains 19. The auxiliary domains include Gly-rich, acidic domains and others. The following table shows the isoforms, molecular weights, and different domains of hnRNPs (Table 1). In summary, the molecular functions of hnRNPs vary depending on their specific domains, RNA recognition motifs (Figure 1), post-translational modification sites and cellular localization.

At DNA level, hnRNPs are involved in DNA replication, transcription, damage repair, chromatin remodeling, telomerase activity and telomere length maintenance. At RNA level, they play important roles in maintaining the stability of mRNA, and participate in RNA splicing. At protein level, hnRNPs can affect protein translation and degradation by binding to other proteins.

In addition to affecting other genes at DNA, RNA and protein levels, hnRNPs can alter their own nucleo-plasmic shuttling, subcellular localization, and metabolism through phosphorylation, ubiquitination, and other post-translational modifications. Post-translational modification (PTM) refers to the chemical modification of a protein after translation. For most proteins, this is a later step in protein biosynthesis. PTM includes protein ubiquitination, phosphorylation, methylation, acetylation, slunoylation and so on. For example, the MAPK/ERK signaling pathway activates hnRNPK phosphorylation at Serines 284 and 353 in hepatocellular carcinoma, leading to the entry of hnRNPK from the nucleus into the cytoplasm and an increase in its protein level in the cytoplasm 20. E3 ubiquitin ligase ZFP91 promotes ubiquitination of oncoprotein hnRNPA1 at lysine 8 and subsequent proteasomal degradation, thereby resulting in inhibition of hnRNPA1-dependent PKM splicing and suppression of glucose metabolism in HCC 21. In addition, the post-translational modification of hnRNPs affects the binding of hnRNPs to certain proteins and RNAs, and regulates mRNA stability 22, 23. hnRNPA2/B1 is regulated by lncRNA molecules. When lncRNA miR503HG and hnRNPA2/B1 form complexes in HCC, hnRNPA2/B1 protein ubiquitination is promoted, the stability of P52 and P65 mRNAs is reduced, and the NF-kappa B pathway of HCC is inhibited 24. The following table contains the different modification sites for hnRNPs (Table 1).

RNA-binding motifs are required for every member of the hnRNP family. Except for hnRNPE, hnRNPK and hnRNPU, all other members have at least one RRM domain. In addition to RRM, KH and RGG domains are also RNA binding motifs, which play a major role in the biological functions of hnRNPs. The Gly rich domain and the acid rich domain belong to auxiliary domains. The auxiliary domains perform together with RNA binding motif to help RNA binding protein to function. Among different hnRNPs, there are both the same and different RNA binding motifs and auxiliary domains. Therefore, the members of hnRNPs share common RNA binding characteristics, but the binding sites are different.

## The functions and the underlying mechanisms of hnRNPs in stem cells

hnRNPs function in stem cells through multiple mechanisms (Table 2). The mechanisms of hnRNPs mainly include: (1) hnRNPs bind directly with mRNAs or form complexes with lncRNAs and mRNAs to regulate mRNA stability. (2) hnRNPs bind with mRNA and induce alternative splicing. (3) hnRNPs affect embryonic development by epigenetic modification. (4) hnRNPs regulate telomerase activity and telomere length in stem cells.

## hnRNPs regulate mRNA stability and gene transcription in stem cells

As typical RNA-binding proteins, hnRNPs play a vital role in mRNA stability and gene transcription regulation. hnRNPs bind with lncRNA in stem cells to form complexes with mRNA or directly bind with mRNA, thus affecting the stability of mRNA. Pnky is a lncRNA presented in embryonic brain with neural-specific functions and regulates neural stem cells (NSCs) in life activities. Knockdown of Pnky leads to the differentiation of NSC in mouse embryonic brain. Polypyrimidine tract binding protein 1 (PTBP1 or hnRNPI) is an effective regulator of the neurogenesis, which has been shown in previous studies 25. Knocking down PTBP1 leads to premature differentiation of NSC in mouse brain and impairs motor behavior 26, while PTBP1 can affect neuronal gene expression by regulating the transcription factor Pbx1 27. Through RNA pull down experiment and RNA immunoprecipitation technique, Pnky and PTBP1 were found to specifically bind to each other. After Pnky and PTBP1 were knocked down respectively, it was found through DEX-Seq 28 sequencing that there were a common part of transcription alterations that affect the differentiation of NSCs and regulate neurogenesis and neuronal development 26. A new study has shown that PTBP1 can bind and stabilize ID2 mRNA by interacting with lncRNA ANCR, thus inhibiting the differentiation of human adipogenic mesenchymal stem cells (hAMSCs) into defined endoderm cells and reducing the occurrence of liver and pancreatic diseases 29.

Most members of the hnRNPs have similar roles because they share the same structural domains and components. It is anticipated that the members may participate in cellular biological processes by binding to each other and forming complexes together. For example, when studying the self-renewal and glycolytic pathways of mouse embryonic stem cells, lncRNA Lncenc1 was found to have the highest expression 30, and through biotin labeling, Lncenc1 was then found to bind to hnRNPK and PTBP1. Previously, it has been proved through yeast two-hybrid technology that hnRNPK and PTBP1 bind to each other 31. Using chromatin immunoprecipitation (ChIP) and chromatin isolation by RNA purification analysis, hnRNPK was found to bind with the promoter of glycolysis gene and directly regulate the transcription of glycolysis gene. When hnRNPK and PTBP1 are knocked down respectively, the glycolysis gene will be down-regulated and lactate production will be reduced. The above results indicate that Lncenc1, hnRNPK and PTBP1 form complexes to promote glycolytic pathway and maintain the self-renewal of mouse embryonic stem cells. PTBP1 is involved in the transcriptional regulation in cells and controls the stability of mRNA. In the development of erythropoiesis, heme, an important component of hemoglobin, is a part of the stabilizing regulators 32, 33. Heme first activates a series of enzymatic reactions through the red cell specific gene 5-aminolevulinic acid synthase 2 (ALAS2, or ALASE) 34, 35, and then it is synthesized in the proerythroblast. In contrast, once ALAS2 gene expression is too low, it will lead to insufficient heme synthesis, resulting in the differentiation of proerythroblast and anemia 36. In the study of erythroid differentiation, it was found that lncRNA UCA1 can act as an RNA scaffold, and specifically bind hnRNPI to recruit ALAS2 mRNA and control its stability. Keeping the transcription and translation of the ALAS2 gene under control for a relatively stable state, quantitative heme can be synthesized to participate in the development of red blood cells 37.

Acute myeloid leukemia (AML) is a malignant transformation of myeloid hematopoietic stem/progenitor cells that can be life-threatening if not detected and treated early 38, 39. At present, it has been found that hnRNPs can regulate mRNA stability of the key factors in leukemia stem cells, thereby affecting the occurrence and development of leukemia. It provides a new therapeutic target for the treatment of leukemia. For example, MSI2 is a critical factor participating in self-renewal of leukemia stem cells (LSCs) 35. With the proteomics analysis of RBP networks in LSC involving MSI2 protein interaction and functional shRNA screening, highly expressed SYNCRIP (or hnRNPQ1) was identified. SYNCRIP indirectly binds to MSI2 and interacts with a common mRNA target, HOXA9, thus maintaining its transcription and translation, inhibiting apoptosis and promoting leukemia 34. Recently, hnRNPD overexpression was found to be involved in the induction of chronic myelogenous leukemia (CML). hnRNPD recognizes the special substrate sequence ACUAGC in PBX1 3'-UTR and binds with it, thus stabilizing PBX1 mRNA, promoting its translation, and allowing the colony-forming cells (CFCs) to produce more CML CD34+ cells, which provides a new treatment strategy for chronic myeloid leukemia 36. Knocking down hnRNPD in CML cells results in down-regulation of PBX1 expression, which inhibits CML cell growth and lead to sensitization of CML cells to imatinib.

Calorie intake has been previously reported to play a role in cellular aging 40, 41. Ketone body β-hydroxybutyrate (β-HB) acts as an intermediate metabolite in fat oxidative metabolism to prevent aging. The direct binding of β-HB to hnRNPA1 was found by using MALDI-TOF mass analysis 42. Oct4, as a stem factor, has an anti-aging effect through its own expression. Knocking down Oct4 causes aging partly because of induced DNA damage. In endovascular cells, the mRNA stability test showed that hnRNPA1, in the presence of β-HB, can enhance the stability of Oct4 mRNA and enable the normal expression of Oct4 and its downstream LaminB1, which is one of the key factors to resist senescence induced by DNA damage 43. Therefore, β-HB can maintain the self-renewal of endovascular cells and delay senescence by up-regulating hnRNPA1 and inducing Oct4-mediated LaminB1 pathway 44.

There are two main causes for the occurrence of diseases such as muscle weakness and muscle atrophy. One is the bone damage caused by strenuous exercise or serious accident, which leads to the damage of skeletal muscle stem cells and myoblast cells and the impairment of muscle regeneration. The other is that as muscle stem cells grow older, their ability to self-renew and reproduce decreases. Chenette et al. argued that AUF1 (or hnRNPD) can act as a regulator in adult muscle stem cells, which is important in skeletal muscle formation 45. Meanwhile, Abbadi et al. found that AUF1 can target and regulate Twist1 mRNA by combining the AU-rich elements (AREs) in the 3'-UTR, which rapidly degrades Twist1 mRNA and leads to the weakening of self-renewal of muscle stem cells and the differentiation of myogenic cells 46. It follows that hnRNPs can play a significant role in skeletal muscle growth and muscle injury repair by regulating the stability of mRNA.

In addition to lncRNA, microRNA, as a kind of non-coding small RNA, can also directly regulate gene post-transcriptional expression or stabilize its own expression by binding to RBPs and functioning together in cells. High expression of PCBP1 (also known as hnRNP-E1 or CP-1) was found in mouse myoblasts, and the hybrid offspring of PCBP1 mutant mice showed fetal death, indicating that PCBP1 plays a crucial role in the growth and development of mice. In 2017, Espinoza et al. found that PCBP1 combines with Argonaute 2 to stabilize miR-1, miR-133, and miR-206 in muscle cells, which contributes to the expression and processing of these miRNAs in myoblasts, inhibits the conversion of fast to slow muscle fibers in muscles, and regulates the contraction and relaxation of skeletal muscles 47.

hnRNPs not only can recruit lncRNA and miRNA to regulate the expression of downstream target genes and affect the stability of their mRNA to participate in the development process of stem cells, but also can maintain the pluripotency of stem cells and prevent premature differentiation by directly binding and degrading mRNA with the function of promoting cell differentiation. Recent results have shown that hnRNPK can prevent premature differentiation of epidermal progenitor cells by directly binding and degrading GRHL3 and KLF4 transcripts, while knocking down hnRNPK leads to cell differentiation 48.

## hnRNPs regulate the alternative splicing of mRNA in stem cells

Alternative splicing (AS) can affect the transcriptional regulation and translation of genes, resulting in different or antagonistic functions of the final protein and different meanings in cell activities 49, 50. mRNA splicing can be used as a gateway to control stem cell pluripotency and differentiation. The generation of AS can be broadly divided into three categories, i.e., exon jump, mutually exclusive exons, and intron retention 51. hnRNPs, a well-known family of splicing proteins, have been widely studied, and great relevant advances in the field of stem cells have been achieved in recent years. For example, hnRNPF/H can modulate the pluripotency of human embryonic stem cells by alternative splicing TCF3. TCF3 (or E2A) is a member of the transcription factor E protein family, all of which are regulated by stem factors Oct4, Nanog and Sox2, and can bind to the promoter of downstream genes that regulate the differentiation of embryonic stem cells. TCF3 possesses two isoforms generated by alternative splicing, E12 and E47, both of which are transcription factors of CDH1. CDH1 can keep mouse ESCs (mESCs) in their naïve state, and interfering with CDH1 results in differentiation of mESCs into ectoderm cells 52, 53. hnRNPF/H predominantly promotes E12 expression in human embryonic stem cells, thus elevating CDH1 protein expression and maintaining pluripotency of human embryonic stem cells. Knockdown of hnRNPF/H causes TCF3 to be spliced into E47 variant with exon 18a skipped and leads to the differentiation of human embryonic stem cells into ectoderm by inhibiting CDH1 expression 54, 55. hnRNPF/H mainly regulates TCF3 alternative splicing by combining with the exon splicing silencer (ESS) in exon 18b of TCF3. Yamazaki et al. found another way by which the alternative splicing of TCF3 is regulated. hnRNPH1 and PTBP1 first combine with each other and then bind together to the conservative intron-spliced silencer (ISS) of TCF3, remotely regulating the variable splicing of TCF3 56.

Amyotrophic lateral sclerosis (ALS), also known as Lou Gehrig's disease, is a progressively increasing and fatal neurodegenerative disease 57. The cause of 90-95% of the disease cases is unknown, and about 5-10% of them are inherited from parents. Most importantly, ALS has no effective cure to date. Martinez FJ et al. found that an RNA network is associated with neurodegeneration, which involves alternative splicing regulated by hnRNPA2/B1. Knocking down hnRNPA2/B1 can cause changed mRNA splicing of ALS-related D-amino acid oxidase (DAO) gene, lead to the skipping of exons in DAO, and change the expression of its downstream target genes. DAO encodes an enzyme involved in D-serine metabolism, which has been reported to play a role in the excitation process of ALS 58. Therefore, knocking down hnRNPA2/B1 will eventually lead to decrease in D-serine metabolism, resulting in ALS disease 59.

Toll-like receptors (TLRs) have a function in human body to regulate myelogenesis, but prolonged activation of TLR causes dysfunction in the production of hematopoietic stem/progenitor cells (HSPCs), leading to malignant blood disorders 60. TNF receptor-associated factor-6 (TRAF6) is an E3 ligase, and acts as a TLR effector with ubiquitin (Ub) ligase activity. Once overexpressed in HSPC, it activates TLR to promote the development of myelodysplastic syndrome 61, 62. By using human leukemia cell lines overexpressing TRAF6, Fang et al. performed a global semi-quantitative Ub screening and a TRAF6 substrate ubiquitin screening, and revealed that as one of the substrates, hnRNPA1 is ubiquitinated by TRAF6. Exon expression microarray analysis showed a high enrichment of exons associated with RNA-binding proteins, and subsequent in-depth studies revealed that TRAF6 leads to jumping of exon 2 of Arhgap1 primarily through ubiquitination of hnRNPA1, resulting in truncated ORF-encoded Arhgap1 protein and increased cdc42 activity, and the ultimate manifestation is myelogenous differentiation and severely hampered hematopoiesis 63.

The maintenance of the body's immune system against viruses is largely dependent on immunoglobulins secreted by plasma cells. Plasma cells are differentiated from B cells and are at the terminal stage of B lymphocytes. By comparing the proteomics of B cells and plasma cells, it has been found that one of the RNA-binding proteins exhibits significant difference. hnRNPLL expression level is lower in B cells, while it increases significantly in plasma cells. In order to investigate the role of hnRNPLL in the differentiation of B cells into plasma cells, Chang X et al. used Photoactivatable-Ribonucleoside-Enhanced Cross-Linking and Immunoprecipitation (PAR-CLIP) 64, 65 and RNA sequencing methods to search for hnRNPLL target genes, and found that hnRNPLL mainly binds with 3'UTR of target mRNAs. It is well known that the 3'-UTR is one of the cis-acting elements that regulates mRNA stability and translation. The expression of target genes is significantly decreased after knocking down hnRNPLL. It follows that the binding of hnRNPLL with 3'-UTR enhances the stability of mRNAs in cells 66. In plasma cells, hnRNPLL regulates the transition from membrane isoform to secreted isoform of immunoglobulin heavy chain through binding to RRM1 domain of PABPC1 in plasma cells, but the exact mechanism remains to be investigated 67.

## hnRNPs epigenetically regulate gene expression to affect embryonic development

The modification of N6-methyladenosine (m6A) is one of the most common modifications involved in mRNA regulation in eukaryotic cells. What's more, it is a current hot-spot research area. The current results indicate that m6A modification plays a crucial role in regulating the life activity of stem cells, which maintains their pluripotency and promotes stem cell transformation 68-70. m6A modification can enhance the interaction of RNAs and RNA-binding proteins and thus function at post-transcriptional level.

As one of the “readers” of m6A, hnRNPs can recognize the methylation sequence on RNAs. The “m6A-switch” mechanism says that m6A regulates the interaction between RNAs and proteins by reconstructing part of RNA structure. For example, hnRNPC binds to m6A-modified RNA through an “m6A-switch” mechanism. When the m6A-mediated “RNA hairpin” is lost, the single-stranded hnRNPC-binding motif is exposed and its function is altered 71. Alternative splicing of mRNA and target gene expression occur upon binding of the low-complexity region at the C-terminal end of the hnRNPG molecule with m6A 72. However, the role of hnRNPA2/B1 in m6A regulator is controversial among different research groups. Since the initial discovery in 2015, hnRNPA2/B1 has been recognized as the m6A “writer”, which can interact with numerous nuclear transcripts and regulate their alternative splicing 73. In 2018, Wu et al. confirmed by bioinformatics combined with experiments that hnRNPA2/B1 can regulate m6A modifications through an “m6A-switch” mechanism, rather than affect them directly 74. In 2019, it was further shown that knocking down hnRNPA2/B1 delays mouse embryonic development. Meanwhile, knock-down of MELLT3, a methyltransferase that catalyzes m6A methylation, can induce developmental defects that prevent the formation of embryos. To further confirm the link among hnRNPA2/B1, m6A, and MELLT3, Immunocytochemistry (ICC) was used to detect the localization of m6A. Knocking down of MELLT3 resulted in reduced hnRNPA2/B1 and m6A levels. It was also found that hnRNPA2/B1 is regulated by METTL3-dependent m6A RNA methylation to maintain the self-renewal in mouse embryonic development 75.

Chromosome inactivation, also known as lyonization, is an epigenetic event at which one of the two X chromosomes in cells of female mammals is concentrated and heterochromatinized due to the inactivation of this X chromosome called inactive X chromosome (Xi) 76. One gene that plays a crucial role in the formation of Xi is X inactive-specific transcript (Xist), whose expression product acts as a long non-coding RNA and participates in epigenetic modification. It induces the formation of heterochromatin by recruiting transcriptional silencing complexes.

hnRNPU (or SAF-A), as one of the proteins forming the nuclear matrix, was found by Hasegawa et al. to interact with Xist RNA via its RGG RNA binding domain, which was confirmed by UV cross-linking analysis. In cultured mouse neuroblastoma cell line Neuro2a and mouse embryonic fibroblasts (MEFs), researchers found by immunofluorescence that after hnRNPU is knocked down, Xist is detached from the Xi, moves toward the nucleoplasm and disperses. hnRNPU-deficient mice exhibit early embryonic lethality after hybridization 77. However, Kolpa et al. expressed inconsistent views. They knocked hnRNPU down in multiple cell lines to verify whether it plays an important role in the enrichment of Xist RNA on Xi chromosome and has generalizability. The results showed consistency with Hasegawa et al. only in the mouse Neuro2a cell line. But in MEF cells and renal cells, Xist RNA was not fully released 78. Basic research permits disputes and queries. Sakaguchi, one researcher of Hasegawa's experimental team, explained that the discrepancy could be due to inefficient hnRNPU knockdown resulted from different vectors, transfection agents, and experimental methods used in other labs. He added more experimental data to prove his team's point 79.

Xist RNA is able to recruit transcriptional silencing complexes to play a role mainly because it possesses a Xist RNA Polycomb-Interaction-Domain (XR-PID), and once the XR-PID is deleted, Xist RNA is unable to recruit those transcriptional silencing complexes and further induce the formation of Xi. Some studies have indicated that knocking down Polycomb group RING finger 3/5 (PCGF3/5-PRC1) can lead to the death of female embryos 80. Researchers have demonstrated that hnRNPK can perform functions similar to XR-PID. Pintacuda et al. found that hnRNPK can bind to XR-PID and recruit transcriptional silencing complex at the same time. Even in the absence of XR-PID, hnRNPK can still help Xist RNA recruit enough transcriptional silencing complexes, though not as efficiently as in the presence of XR-PID 80. This finding provides a new perspective on the role of interactions between non-coding RNA and RNA-binding proteins in chromatin modification.

## hnRNPs regulate telomerase activity and telomere length of stem cells

Telomeres are located at the end of eukaryotic chromosomes and play a major role in protecting chromosomes, preventing chromosome degradation and stabilizing genomes. The length of telomeres is regulated by telomerase, a special DNA polymerase. In general, the presence of active telomerase can only be detected in embryonic stem cells and pluripotent stem cells, which can prolong the damaged telomere and increase the number of cell division, and is also the fundamental cause for the continuous division ability of embryonic stem cells and pluripotent stem cells 81-83.

It is well known that hnRNPs belong a typical RNA-binding protein family, but due to thier RRM domains, hnRNPs can not only bind RNA, but also have the ability to bind DNA. Some hnRNPs can bind telomere DNA to regulate telomerase activity, and some can directly bind telomerase to affect cell function 15, 84.

hnRNPF/H is mostly expressed in undifferentiated stem cells, including embryonic stem cells, which are in a naïve state, and in adult stem cells such as human mesenchymal stem cells (hMSC). hnRNPF/H has been mainly studied as a shear factor, which can regulate the stability of mRNA, and is involved in gene transcriptional process of cells. A recent study indicated that after knocking down hnRNPF/H, the proliferation of mesenchymal stem cells is slowed down and even cell senescence occurs. It has been shown that the growth of MSCs is closely related to the length of telomeres and the activity of telomerase 85, 86. With further differentiation of MSCs into chondrocytes, the length of telomeres gets shorter and the activity of telomerase decreases. To explore whether hnRNPF/H knockdown induced aging of MSCs is related to telomeres and telomerase activity, Xu et al. performed biotin RNA pull-down and RNA-IP experimental studies, and revealed that hnRNPF/H binds to the 5'-end of human telomerase RNA component (hTERC), one of the components of telomerase, and the first three G regions of hTERC bind with the RRM1 domain of hnRNPF/H 87. hTERC and human telomerase reverse transcriptase (hTERT) are important components of telomere formation complex. Using the hTERC as a template, the telomerase DNA is added to the ends of chromosomes to maintain telomere length, which is catalyzed by hTERT 88. After overexpression of hnRNPF/H, the proliferative capacity of mesenchymal stem cells is enhanced. The revealed new role of hnRNPF/H in the regulation of telomeres and telomerase in stem cells brings new directions and ideas for future stem cell research 87.

## Conclusions and perspectives

**Figure 2:**
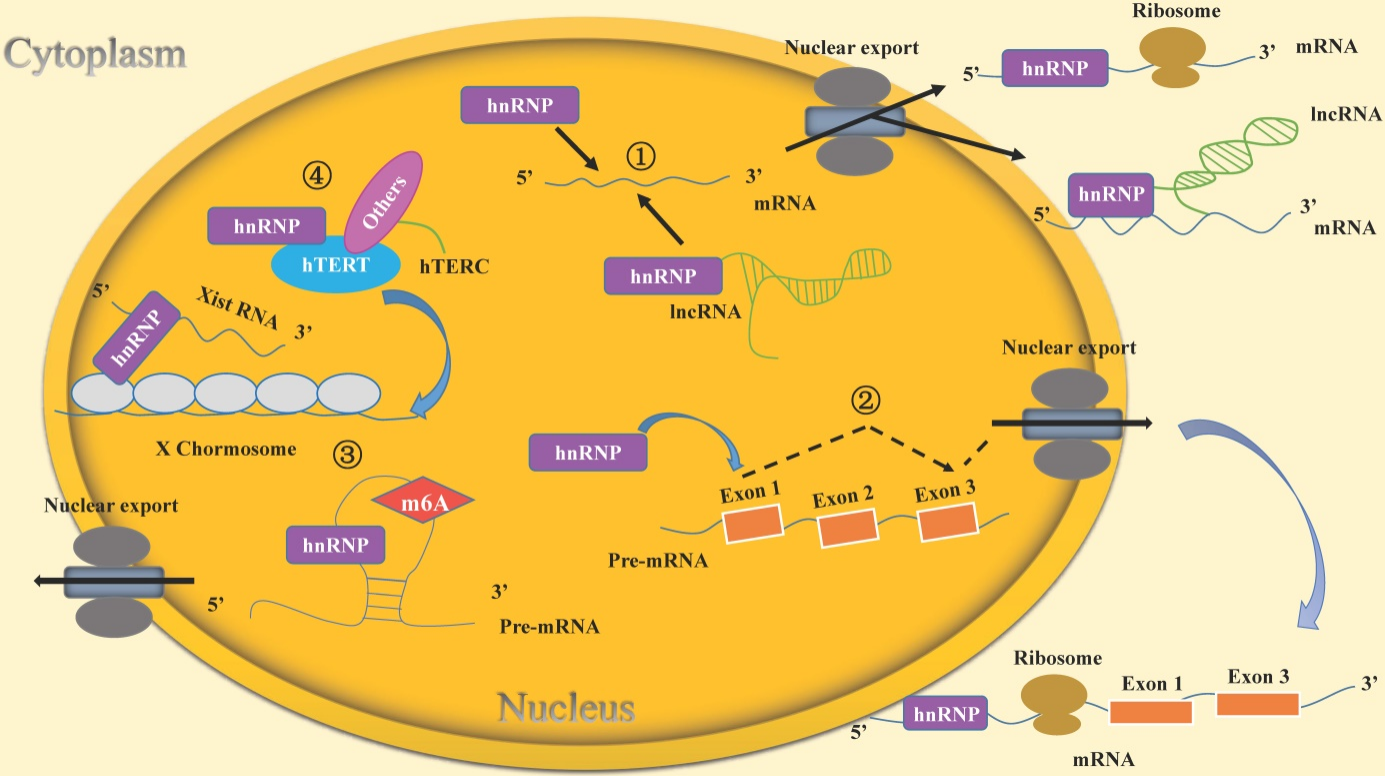
** Molecular mechanisms of hnRNPs included in the life cycle of stem cells.** (1) hnRNPs bind directly to mRNAs or lncRNAs to regulate mRNA stability. For example, hnRNPI binds to the lncRNA Pnky and then interacts with the mRNA of ID2 to regulate its stability. (2) hnRNPs induce the alternative splicing of mRNAs. For instance, hnRNPF/H can regulate alternative splicing of TCF3, resulting in two isoforms E12and E47 that play different roles in the life cycle of stem cells. (3) hnRNPs epigenetically regulate gene expression to affect embryonic development. hnRNPs affect the transcription of surrounding mRNAs through m6A modification. Their own expression is also regulated by this kind of RNA modification. For example, hnRNPA2/B1 is regulated by METTL3-dependent m6A methylation to maintain self-renewal of stem cells in mouse embryo development. Moreover, hnRNPs bind with Xist RNA and participate in chromatin modifications. For example, hnRNPK recruits a transcriptional silencing complex with Xist RNA to induce the formation of heterochromatin. (4) hnRNPs regulate telomerase activity and telomere length in stem cells. For example, hnRNPF/H forms a multimeric complex with hTERC, which contributes to telomere addition to chromosome ends and maintenance of telomere length.

In this review, we have described the mechanisms by which the RNA-binding protein hnRNPs maintain the self-renewal capacity of stem cells and participate in the stem cell differentiation process. The main mechanisms include the followings: 1. hnRNPs regulate the stability of mRNA in stem cells. 2. hnRNPs regulate alternative splicing of mRNAs in stem cells. 3. hnRNPs epigenetically regulate gene expression and affect embryonic development. Firstly, hnRNPs are involved in regulation of m6A modification to affect the transcription of surrounding mRNAs and thus maintain stem cell self-renewal. Secondly, hnRNPs bind with Xist RNA and are involved in chromatin modification. 4. hnRNPs regulate telomerase activity and telomere length in stem cells (Figure 2).

Although some progresses have been made in the research field of hnRNPs, the functions played by hnRNPs in stem cells deserve further investigation. To date, many achievements and breakthroughs have been obtained about the roles of hnRNPs in the occurrence and development of malignant tumors. For example, hnRNPs play a role in inhibiting epithelial-mesenchymal transition (EMT) 89, and in cell invasion and apoptosis through TGF-β 90, 91, AKT 92, p53 93, 94, and other signaling pathways, suggesting that hnRNPs may be used as novel therapeutic targets. In addition to family heredity, somatic gene mutations, influence of external environment and uncontrolled self-renewal capacity of the body's stem cells contribute a lot to the occurrence of malignant tumors and other diseases. Therefore, the study of the activities of stem cells in the organism is of great scientific significance. However, there are relatively less studies on the mechanism of hnRNPs in stem cells compared to cancers at present. Meanwhile, in the existing stem cell studies, the majority of the individual hnRNP family members have been investigated through their interactions with RNAs and other proteins. Rare exploration is focused on the interactions among hnRNP members in biological processes of stem cells, which is likely to reveal new biological functions of hnRNPs. It is interesting that some hnRNPs can function by binding to the same RNA molecule. The combination of L2 mRNA with hnRNP E1, E2, and K inhibits the translation of human papillomavirus type 16 95. The interactions among hnRNPs members have been detected by the yeast two-hybrid system and *in vitro* co-precipitation assays. The results showed that hnRNPE2 and hnRNPK can bind with each other through their KH domains, and the RRM2 domains in hnRNPI and hnRNPL play a crucial role in their protein-protein binding 30. In addition to binding to each other through their specific domains, the interaction among hnRNPs is also promoted by their PTMs, such as phosphorylation. For example, when studying the mechanism of tumors, it was found that vascular endothelial growth factor (VEGF)-A generated by cells in the process of hypoxia and inflammation is an important cause of angiogenesis. Yao et al. found that hypoxia can induce hnRNPL phosphorylation at Tyr359, which promotes its combination with hnRNPA2B1, and at the same time, phosphor-hnRNPL recruits DRBP76 (double-stranded RNA binding protein 76) to bind to 3'UTR of VEGFA. This unique HILDA (hypoxia-inducible hnRNPL-DRBP76-hnRNPA2/B1) complex allows VEGFA to be stably translated during hypoxia and inflammatory processes 96. This discovery suggests that hnRNPs can bind to each other through phosphorylation or other post-translational modifications and work together in cells. These results bring a new perspective for future research on how different hnRNP members work together to regulate the biological processes of stem cells.

## Figures and Tables

**Table 1:** Structural and functional characteristics of the hnRNPs

hnRNPs (Aliss)	Isoform	Molecular weight (kDa)	RNA-binding domain	Modification sites	Location
hnRNP A/B	A0, A1, A2/B1, A3	34-40	2×RRM, Gly rich, RGG	Methylation site: Arg-266; Sumoylated in exosomes	Nucleus, cytoplasm
hnRNPC	C1, C2	41/43	RRM, acid rich	Phosphorylation site: Ser-260, Ser-299; hnRNP C1 is modified by SUMO at lysine 237	Nucleus
hnRNPD (AUF1)		44-48	2×RRM	Dimethylation site: Arg-345	Nucleus, cytoplasm
hnRNPE	E1, E2, E3, E4	35-40	3×KH		Nucleus, cytoplasm
hnRNPF		53	3×qRRM, 2×Gly rich	Sumoylated	Nucleus
hnRNPG (RBMX)		43	RRM, Gly rich		Nucleus
hnRNPH	H1, H2, H3	50-60	3×qRRM, 2×Gly rich		Nucleus
hnRNPI (PTBP1)		59	4×RRM		Nucleus, cytoplasm
hnRNPL		68	4×RRM, Gly rich	Phosphorylationsite: Ser-544;	Nucleus
hnRNPK (AUKS)		55-65	3×KH, other	Dimethylation site: Arg-296, Arg-299; Sumoylated by CBX4; Ubiquitinated by MDM2; O-glycosylated (O-GlcNAcylated)	Nucleus, cytoplasm
hnRNPM (CEAR)		77	3×RRM	Sumoylated	Nucleus
hnRNPQ (SYNCRIP)	Q1, Q2, Q3	55-70	3×RRM, acid rich	Phosphorylated on tyrosine.	Nucleus
hnRNPR		71	3×RRM, acid rich, RGG, other		Nucleus
hnRNPU (SAF-A)		120	Acid rich, Gly rich, RGG, other	Extensively phosphorylated; Dimethylation site: Arg-739.	Nucleus
hnRNPP2 (FUS/TLS)		72	2×Gly rich, RRM, 2×RGG	Dimethylation site: Arg-216, Arg-218; Phosphorylationsite: N-terminal region	Nucleus

**Table 2:** Molecular functions and mechanisms of hnRNPs in stem cells

hnRNPs (Aliss)	Bound RNA	Expression	Function	Mechanism
hnRNPI (PTBP1)	Pnky, ANCR	High	Inhibit the differentiation of stem cells	Maintain mRNA stability 28
hnRNPI (PTBP1)	UCA1	High	Involved in the development of red blood cells	Maintain mRNA stability 36
hnRNPQ1 (SYNCRIP)	MSI2	High	Maintain the self-renewal capacity of leukemia stem cells	Maintain mRNA stability 33
hnRNPA1 (ALS19)	β-HB	High	Maintain self-renewal of blood vessel cells and delay aging	Maintain mRNA stability 43
hnRNPD (AUF1)	Twist1	High	Promote myoblast differentiation	Degrade mRNA 22,43
hnRNPE1 (Pcbp1)	Argonaute 2	High	Inhibit myoblast differentiation	Maintain mRNA stability 46
hnRNPK	GRHL3, KLF4	High	Inhibit epidermal progenitor cell differentiation	Degrade mRNA 47
hnRNP F/H	TCF3	Low	Differentiation of human embryonic stem cells into the outer germ layer.	Alternative splicing of mRNA 51-55
hnRNP A2/B1	D-amino Acid Oxidase	Low	Induce the occurrence of ALS disease	Alternative splicing of mRNA 57,58
hnRNPA1 (ALS19)	TRAF6	High	Bone marrow differentiation	Alternative splicing of mRNA, exon skipping 62
hnRNPLL	PABPC1	High	Promote the differentiation of B cells into plasma cells.	Alternative splicing of mRNA 66
hnRNP A2/B1		High	Maintain self-renewal in mouse embryonic development	M6A modification 72-74
hnRNPU (SAF-A)		Low	Mouse embryonic lethal	X chromosome inactivation 77,78
hnRNPK		Low	Mouse embryonic lethal	X chromosome inactivation 80
hnRNP F/H		High	Maintain telomere length and telomerase activity of stem cells	Binding to the 5'-end of hTERC 87

## Abbreviations

hnRNPs: the heterogeneous nuclear ribonucleoproteins
ESCs: embryonic stem cells
ASCs: adult stem cells
RBPs: RNA-binding proteins
RBD: RNA-binding domains
ORF: open reading frame
UTR: untranslated region
KH: K homology
dsRBM: double-stranded RNA-binding base sequence
ZF: zinc finger
RRM: RNA recognition motif
PTM: post-translational modification
NSCs: neural stem cells
PTBP1: polypyrimidine tract binding protein 1
hAMSCs: human adipogenic mesenchymal stem cells
ChIP: chromatin immunoprecipitation
ALAS2: aminolevulinic acid synthase 2
AML: acute myeloid leukemia
LSCs: leukemia stem cells
CML: chronic myelogenous leukemia
CFCs: colony-forming cells
β-HB: β-hydroxybutyrate
AREs: AU-rich elements
AS: alternative splicing
mESCs: mouse ESCs
ESS: exon splicing silencer
ISS: intron-spliced silencer
ALS: amyotrophic lateral sclerosis
DAO: D-amino acid oxidase
TLRs: Toll-like receptors
HSPCs: hematopoietic stem/progenitor cells
TRAF6: TNF receptor-associated factor-6
Ub: ubiquitin
PAR-CLIP: Photoactivatable-Ribonucleoside-Enhanced Cross-Linking and Immunoprecipitation
m6A: N6-methyladenosine
Xist: X inactive-specific transcript
MEFs: mouse embryonic fibroblasts
XR-PID: Xist RNA Polycomb-Interaction-Domain
PCGF3/5-PRC1: Polycomb group RING finger 3/5
hMSC: human mesenchymal stem cells
hTERC: human telomerase RNA component
hTERT: human telomerase reverse transcriptase
VEGFA: vascular endothelial growth factor (VEGF)-A
